# Seizures as the initial manifestation: characterizing the adult phenotype of MOG antibody-associated disease

**DOI:** 10.3389/fneur.2026.1746963

**Published:** 2026-02-24

**Authors:** XuXin Cao, Teng Wang, Pei Zhang, JuXian Kang

**Affiliations:** 1 Department of Neurology, Graduate School of Chengde Medical University, Chengde, Hebei, China; 2 Department of Neurology, Xingtai People’s Hospital, Xingtai, Hebei, China; 3 Department of Rehabilitation Medicine, Xingtai People’s Hospital, Xingtai, Hebei, China

**Keywords:** adult clinical features, cortical signal abnormalities, epileptic seizures, immunotherapy, myelin oligodendrocyte glycoprotein antibody-associated disorders

## Abstract

**Objective:**

Clinical Characteristics of Adult MOG Antibody-Associated Disease Presenting with Seizures as the Initial Symptom.

**Methods:**

A retrospective analysis was conducted on the clinical data of 7 patients who presented with epileptic seizures as the initial symptom among 16 adult MOG antibody-positive patients admitted to the Department of Neurology at Xingtai People’s Hospital in Hebei Province from August 2020 to March 2025.

**Results:**

In this study, the age of onset of the 7 patients ranged from 18 to 75 years. All patients presented with epileptic seizures as the initial symptom, with 3 cases accompanied by fever and 4 cases by headache. Among the 7 patients, seizure types included focal seizures and generalized tonic–clonic seizures. Cerebrospinal fluid (CSF) pressure was elevated in 4 cases, while leukocyte count was increased in 2 cases. Cranial magnetic resonance imaging (MRI) revealed cortical lesions in 3 patients without significant meningeal enhancement, and 4 cases showed no abnormal findings on MRI. Electroencephalography (EEG) revealed focal slow waves in 5 cases, while 2 cases showed normal findings. All 7 patients were treated with hormones, 2 received intravenous human immunoglobulin, and 5 were administered antiepileptic drugs. All 7 patients achieved favorable prognosis, with one case of recurrence.

**Conclusion:**

In adult MOG antibody-positive patients presenting with epileptic seizures as the initial symptom, the disease can occur at any age without a clear sex predominance. These patients may present with non-specific clinical features such as fever, headache, and abnormalities in cerebrospinal fluid and electroencephalography. Some patients may exhibit isolated epileptic seizures. Cranial magnetic resonance imaging often reveals cortical signal abnormalities. For adult patients presenting with seizures as the first symptom, early MOG antibody testing is essential for a definitive diagnosis. Prompt initiation of corticosteroid or intravenous immunoglobulin therapy can effectively control disease progression.

## 1 Introduction

Myelin oligodendrocyte glycoprotein (MOG) is a protein expressed on the surface of myelinating oligodendrocytes in the central nervous system (CNS) (1). MOG antibodies mediate cytotoxicity against MOG-expressing cells through multiple mechanisms (2), leading to an inflammatory demyelinating CNS disorder termed MOG antibody-associated disorders (MOGAD), characterized by the presence of MOG autoantibodies. MOGAD represents a distinct disease spectrum separate from multiple sclerosis (MS) and neuromyelitis optica spectrum disorder (NMOSD). MOGAD lesions can extensively involve the CNS, presenting with diverse clinical manifestations including optic neuritis, meningoencephalitis, brainstem encephalitis, and myelitis. These may occur as a single symptom or in various combinations of the above (3). Previous studies have extensively summarized the clinical and imaging characteristics of MOG antibody-associated diseases. In recent years, case reports of MOGAD and its cortical encephalitis clinical phenotype have garnered significant scholarly attention, with seizures potentially constituting a component of MOGAD’s clinical presentation. Patients with seizures in MOG antibody-associated diseases are increasingly being identified; however, studies on adult MOG antibody-associated diseases presenting with seizures as the initial symptom remain scarce, and their clinical manifestations and imaging features remain uncertain. The prevalence of MOGAD is approximately 1.3–2.5 per 100,000, with an annual incidence of about 3.4–4.8 per 100,000 (4). A domestic meta-analysis (5) reported a seizure incidence of 20.5% in MOGAD. Furthermore, patients with cortical encephalitis and acute disseminated encephalomyelitis (ADEM)-like phenotypes exhibit a higher risk of seizures, with 37.3% experiencing epileptic episodes. Compared to patients with multiple sclerosis and AQP4-NMOSD, seizures are more common in MOGAD patients (5, 6). An increasing number of individuals recognize that epileptic seizures may occur concurrently with or precede demyelinating events, representing a unique clinical feature of MOGAD, mainly accompanied by epileptic seizures with or without brain disorders (7). The precise etiology of seizures in MOGAD patients remains unclear. This study retrospectively analyzed 7 cases of adult MOGAD patients presenting with seizures as the initial manifestation, investigated their clinical features to facilitate early diagnosis and treatment of the disease, and a systematic review of previous cases presenting with FLAMES was conducted.

## 2 Materials and methods

### 2.1 Study population

A retrospective study was conducted on 7 adult patients with MOGAD who presented with epilepsy as the initial symptom among 16 adult MOGAD patients diagnosed and treated in the Department of Neurology at Xingtai People’s Hospital, Hebei Province, from August 2020 to March 2025. The cohort comprised 3 males and 4 females. Inclusion criteria for the 7 patients were: Compliance with the 2023 MOGAD International Expert Panel diagnostic criteria (3): (a) At least one core clinical demyelinating event, including optic neuritis, myelitis, acute disseminated encephalomyelitis, focal or multifocal cerebral lesions with neurological deficits, or cortical encephalitis with seizures; (b) Serum MOG-IgG positivity during the acute phase or at the time of clinical relapse, with or without cerebrospinal fluid (CSF) MOG-IgG positivity; (c) Serum AQP4-IgG antibody negativity; (d) Other diseases such as multiple sclerosis are reasonably excluded. Seizure classification follows the 2017 International League Against Epilepsy (ILAE) classification of seizure types and epilepsy. Exclusion criteria: (a) Incomplete clinical data documentation; (b) Seizures caused by other etiologies; (c) Refusal to sign informed consent.

### 2.2 Research methods

Basic information (age, gender), clinical manifestations, seizure types and frequency, presence of status epilepticus, imaging findings, cerebrospinal fluid and MOG antibody test results, electroencephalogram (EEG) findings, initial diagnosis, treatment regimens (corticosteroids and immunosuppressants, antiepileptic drugs), and prognosis. Lumbar puncture was performed in the patient within 24–72 h after the epileptic seizure. For statistical analyses,using SPSS 27.0 statistical software. Measurement data that conform to the normal distribution are expressed as x ± s, and those that do not conform to the normal distribution are expressed as the median. Counting data are expressed as the number of cases and percentage [*n* (%)].

## 3 Results

### 3.1 General characteristics

Among the 7 MOGAD patients, all had an acute or subacute onset (<3 months), including 3 males (43%) and 4 females (57%), with an onset age ranging from 18 to 75 years, a mean age of 35 years, and a median age of 22 years. The median interval from onset to diagnosis of MOGAD was 7 days. Two MOGAD patients were initially diagnosed with viral encephalitis. Results are summarized in Table 1.

**Table 1:** Comparison of clinical characteristics of 7 adult patients with MOG antibody-associated diseases and epileptic seizures.

Case	Sex	Age (years)	Types and frequencies of epileptic seizures	Initial diagnosis	Other clinical symptoms	Time from onset to diagnosis of MOGAD	Immunotherapy	Pharmacotherapy for epilepsy	Head MRI	Follow-up duration/Month
1	F	43	Two comprehensive ankylosis sessions	Seizure	–	4 days	IVIG	Lacosamide	The left parietal cortex is slightly swollen	6
2	F	75	Two comprehensive ankylosis sessions	Symptomatic Epilepsy	Transient limb movement disorder	4 days	Methylprednisoione Pulse Therapy	Sodium Valproate	–	8
3	F	45	Four times at the stove Four comprehensive ankylosis sessions	Symptomatic Epilepsy	–	10 days	Methylprednisoione Pulse Therapy	Sodium Valproate Levetiracetam	–	47
4	M	22	Set the stove once Two comprehensive ankylosis sessions	Viral Meningitis	Fever headache	7 days	Methylprednisoione Pulse Therapy	–	–	52
5	M	22	Set the stove once One comprehensive rigidity session	Seizure	Headache numbness of limb	10 days	Methylprednisoione Pulse Therapy	Levetiracetam	–	56
6	F	18	Set the stove once	Symptomatic Epilepsy	Fever headache	7 days	Methylprednisoione Pulse Therapy	Levetiracetam	Abnormal signals in the left temporal lobe and occipital lobe	2
7	M	20	Two comprehensive ankylosis sessions	Viral Meningitis	Headache	6 days	IVIG	Sodium Valproate	Abnormal signals in the local cortical area of the left temporal lobe	32

F female, M male, IVIg Intravenous immunogiobulins.

### 3.2 Clinical manifestations

All 7 patients presented with epileptic seizures as the initial symptom. Four cases (57%) exhibited focal seizures as the primary presentation, with 6 (85%) of these being generalized tonic–clonic seizures. Within 24-48 h following epileptic seizures, fever occurred in 3 cases (43%), headache in 4 cases (57%), transient limb movement impairment in 1 case (14%), and limb numbness in 1 case (14%). Cortical FLAIR hyperintensities were detected on cranial magnetic resonance imaging in 4 cases (57%). Among the total 20 epileptic seizures, 1 case (5%) experienced 1 seizure, 6 cases (30%) had 2–4 seizures, 7 (35%) were focal seizures, and 11 (55%) were generalized seizures. Results are summarized in Table 1.

### 3.3 Ancillary examination results

**Figure 1:**
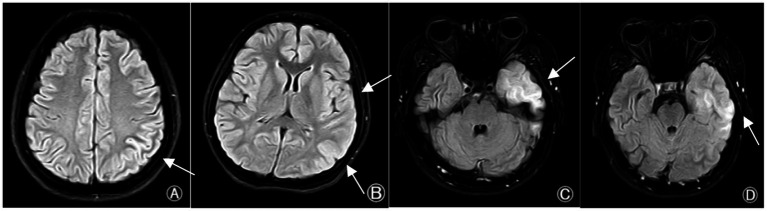
Cranial MRI findings in the acute phase of MOGAD with seizures as the initial symptom in adults. **(A)** In Case 1, there is slight swelling of the left parietal lobe cortex. **(B)** In Case 6, there are abnormal FLAIR hyperintensities in the left temporal lobe and occipital lobe. **(C,D)** In Case 7, there are FLAIR hyperintensities in the local cortex of the left temporal lobe.

(1) CSF Examination: A total of 11 CSF tests were performed in 7 patients within 24 h after epileptic seizures. Elevated initial CSF pressure was found in 3 cases (27%), with the pressure ranging from approximately 190 to 330 mmH₂O; elevated white blood cell count was observed in 3 cases (27%), with the highest count reaching 300cells/μL; elevated protein was detected in 2 cases (18%), with the highest level of 0.546 g/L; no abnormalities were found in glucose and chloride levels; positive CSF MOG antibodies were identified in 4 cases (57%), negative in 3 cases (43%), and the remaining autoimmune antibodies were negative. Positive serum MOG antibodies were found in 6 cases (85%), and negative in 1 case (14%). Results are summarized in Table 2. (2) Cranial MRI: All 7 patients underwent cranial MRI examination in the acute phase, of which 3 cases (43%) showed abnormalities, mainly manifested as abnormal signals in the cerebral cortex, hyperintensities on T2FLAIR in the involved areas, with the lesions presenting as punctate or patchy shapes, and some lesions were swollen. The lesions involved the temporal lobe in 2 cases (28%), parietal lobe in 1 case (14%), and occipital lobe in 1 case (14%) (Figure 1), while 4 cases (57%) were normal. (3) Spinal MRI: All results were normal. (4) Electroencephalogram (EEG): Video EEG examinations were performed in all 7 patients, of which 5 cases (71%) showed abnormal findings, including slow background activity in 3 cases (43%) and focal epileptiform discharges in 2 cases (28%). Results are summarized in Table 1.

**Table 2:** Laboratory test results of 7 adult MOGAD patients with seizures.

NO.	CSF analysis						Blood routine [white blood cells (×10^9^/L)]	Serum MOG-IgG
	Pressure (mmH_2_O)	Leukocyte (cells/μL)	Mononuclear cell (%)	Albumen (g/L)	Cytological results	MOG-IgG		
1	195	2	2	0.276	Normal	−	8	+
2	150	4	2	0.436	Normal	−	6	+
3	190	0	0	0.282	Normal	−	9	+
4	330	123	90	0.546	Abnormal	+	15.12	−
5	140	18	0	0.316	Abnormal	+	10	+
6	145	1	1	0.223	Abnormal	+	10.4	+
7	220	300	89	0.364	Abnormal	+	14.6	+

Cerebrospinal Fluid (CSF) White Blood Cell (WBC) Reference Value: Adults < 8 cells/μL, CSF Protein Reference Value: 0.15–0.45 g/L, Peripheral Blood White Blood Cell (WBC) Reference Value: 3.5–9.5 × 10^9^/L, Erythrocyte Sedimentation Rate (ESR) Reference Value: 0–20 mm/h, CSF, + yes, – no.

### 3.4 Treatment and follow-up

(1) Acute Phase Treatment: All 7 patients (100%) were treated with intravenous methylprednisolone at a dose of 500 mg/day in the acute phase, with the dose gradually reduced after 3–5 days, followed by a switch to prednisone 60 mg once daily. Among them, 2 patients (28%) received intravenous human immunoglobulin at a dose of 0.4 g/(kg·d) for 5 consecutive days. Six patients (85%) were treated with oral antiepileptic drugs (valproate sodium, levetiracetam, and lacosamide, respectively); the antiepileptic drugs were gradually tapered and discontinued 6–12 months after the disappearance of epileptic seizures and absence of active lesions on cranial MRI. (2) Remission Phase Treatment: All 7 patients (100%) took a low dose of prednisone orally after discharge, with the dose gradually reduced slowly, and the tapering process of oral corticosteroids lasted for ≥ 6 months. All patients took the medication regularly and no adverse reactions occurred. (3) Follow-up: The follow-up duration for the 7 patients ranged from 2 to 56 months. One patient (14%) relapsed 5 months after the onset of the disease, and the symptoms were relieved after re-treatment with intravenous human immunoglobulin at a dose of 0.4 g/(kg·d) for 5 consecutive days, with no recurrence during 12 months of follow-up; 6 patients (85%) had no epileptic seizures at discharge or during telephone follow-up (see Table 1).

## 4 Discussion

MOGAD is a unique demyelinating disease of the central nervous system, characterized by the existence of pathogenic antibodies against MOG. This disorder exhibits specific clinical manifestations and unique characteristics, with clinical courses ranging from monophasic to relapsing and a wide spectrum of clinical phenotypes. It is noteworthy that disease presentations differ significantly depending on the age group of patients. Multiple studies have demonstrated that clinical phenotype distribution differs between adult and pediatric MOGAD, with the incidence being higher in the pediatric population than in adults (8). Hor and Fujihara (4) reported that regarding the age-specific incidence of MOGAD (with a mean age of onset of approximately 30 years), approximately 30% of cases occur in the pediatric age group; the prevalence of MOGAD is approximately 1.3–2.5 per 100,000 population, and the annual incidence is approximately 3.4–4.8 per 100,000 population. With increasing awareness of the disease and easier access to MOG antibody testing, the prevalence is expected to rise in the future.

Seizures represent a clinical manifestation of central nervous system inflammatory demyelinating diseases, usually emerging during the acute stage. Notably, seizures as the presenting symptom in adult MOGAD are extremely rare, whereas such seizures are comparatively infrequent in MS and NMOSD. Among pediatric MOGAD cases, the male-to-female ratio generally falls between 1:1 and 1:2, with a median seizure onset age of 6 years (range 2–14 years) and a seizure incidence of 10.5–18.6% (9). One study (10) indicated that seizures were the predominant symptom in 88.9% of 236 pediatric multifocal encephalopathy cases, mostly manifesting as focal seizures, while a minority of patients developed status epilepticus. These findings align with those reported by Budhram et al. (11). In a study of the Han population in northern China, acute seizures occurred in 21.3% of MOGAD patients, a rate significantly higher than the 0.4% observed in NMOSD patients (12). Retrospective data have shown that 20.5% of MOGAD patients experience seizures, with elevated seizure risk noted in those with cortical encephalitis and acute disseminated encephalomyelitis (ADEM)-like phenotypes (5). As MOGAD incidence increases, the pathogenic mechanisms of adult-onset MOGAD with seizures as the presenting symptom remain poorly elucidated. A study by Wang et al. (13) demonstrated the presence of fluid-attenuated inversion recovery (FLAIR) hyperintense lesions in MOG antibody-associated cortical encephalitis with seizures (FLAMES). FLAIR imaging of FLAMES patients revealed cortical abnormalities most frequently located in the frontal lobe (58.8%), parietal lobe (70.6%), and temporal lobe (64.7%). Common seizure types included focal-to-generalized tonic–clonic seizures (28.6%) and tonic–clonic seizures of unknown origin (38.1%). Along with the growing recognition of MOGAD, case reports of cortical encephalitis with seizures as the main manifestation have gradually increased (14). As described by Budhram et al. (15), cortical encephalitis presenting with seizures manifests clinically as headache, fever and seizures, with typical imaging findings showing hyperintensities on fluid-attenuated inversion recovery (FLAIR) sequences in unilateral or bilateral cortical regions on brain magnetic resonance imaging (MRI), and white matter involvement is generally absent. The pathological features of FLAMES include mild inflammatory changes in the cortex and subcortex without obvious demyelination (16). Typically, cerebral cortical injury induced by seizures presents as hyperintensity on diffusion-weighted imaging (DWI); transient focal hyperintensity on DWI sequences accompanied by decreased apparent diffusion coefficient (ADC) is a characteristic finding of cytotoxic edema. In contrast, in patients with MOGAD-associated cortical encephalitis complicated by seizures, hyperintensity is observed on T2/FLAIR sequences of brain MRI, indicating interstitial edema (17). In the present study, all 7 patients had seizures as their presenting symptom, with 3 cases complicated by fever and 4 by headache. Abnormal brain MRI findings were noted in 3 patients, manifesting as cortical hyperintensities on T2/FLAIR sequences; lesions were punctate or patchy in morphology, with partial lesion swelling, involving the temporal lobe (*n* = 2), parietal lobe (*n* = 1), and occipital lobe (*n* = 1). Cortical FLAIR hyperintensities on brain MRI are helpful for identifying suspected MOGAD-related cortical encephalitis. Literature retrieval (11, 13, 16, 18, 19) showed that most MOG antibody-positive cortical encephalitis patients had seizures, among whom some patients presented with fever and/or headache; several patients were initially misdiagnosed with central nervous system infection, similar to two cases in our present study. Upon admission, these two cases were diagnosed as “viral meningoencephalitis” according to their medical history but failed to respond to symptomatic treatments like antiviral and anti-inflammatory regimens. Thus, we highlight to clinicians that many febrile patients with MOG antibody-associated disease may be underdiagnosed, as cellular-based autoantibody assays are ineffective for their immunological screening. Additionally, MOG antibody screening is likely to be omitted in patients lacking specific clinical features, such as Cases 4 and 7.

Seizures may present alone. Foiadelli et al. (20) documented that focal seizures can occur in patients with unremarkable brain MRI, which predicts the development of classic MOG antibody-associated demyelination in the following days or months. Notably, 4 patients in the present study had seizures as their presenting symptom, with 2 developing headache and fever within 24–48 h post-onset; no lesions were identified on brain MRI, a finding rarely described or summarized in prior literature. We therefore recognize both the uniqueness and rarity of this clinical phenotype, while noting that its correlation with disease severity and clinical outcomes remains undefined. Further multicenter data are required to validate its clinical significance and investigate the potential mechanisms of negative brain MRI—such as ultra-early undetectable lesions, predominant functional impairment, or subtle lesions undetectable by current imaging modalities. These questions all deserve further in-depth exploration. Additionally, we urge clinicians to recognize such neuroimmune-associated manifestations without positive imaging findings to minimize underdiagnosis. Clinicians should be vigilant that demyelinating events or encephalitis can develop following seizures, even after a prolonged interval post-seizure; according to Ramanathan and Yılmaz, these seizures initially manifest as unexplained episodes (21, 22). As is widely recognized, the immune system exerts a key role in epileptogenesis. Liu et al. (9) found that TPO antibody levels increase markedly during epileptic seizures; while these antibodies may be involved in the development and recurrence of immune-associated seizures, they lack specificity as biomarkers for MOGAD. As described by Ogawa et al. (23), seizures are extremely common, and MOG-IgG itself may have no obvious association with unilateral cortical encephalitis complicated by seizures; instead, autoimmune disease and MOG-IgG may coexist. Studies have confirmed that anti-MOG antibodies can overlap with other autoimmune antibodies, such as coexistence with anti-N-methyl-D-aspartate receptor (NMDAR) antibodies (24). MOG antibody-associated cerebral cortical encephalitis patients presenting with seizures show favorable responses to corticosteroid therapy and excellent outcomes (18, 23). Conversely, long-term antiepileptic drug (AED) treatment does not significantly decrease the incidence of acute seizures or epilepsy in terms of preventing recurrent seizures or *de novo* epilepsy (12).

The present study has several limitations. Firstly, it was a retrospective study with a small sample size of merely 7 patients. Secondly, it remains unclear whether seizures are isolated episodes or secondary to cortical encephalitis in adult MOGAD cases presenting with seizures as the initial manifestation. Future prospective, large-scale, multicenter cohort studies are required to systematically characterize this subset of adult MOGAD patients.

## Data Availability

The original contributions presented in the study are included in the article/supplementary material, further inquiries can be directed to the corresponding author.
